# Supplementary material for: Structural basis for effector protein recognition by the Dot/Icm Type IVB coupling protein complex
Source: Nat Commun. 2020 May 26;11:2623. doi: 10.1038/s41467-020-16397-0 (PMC7251119; doi:10.1038/s41467-020-16397-0)
Supplement: Supplementary file 2 — Supplementary Information [file 41467_2020_16397_MOESM2_ESM.pdf]

Supplementary Information

Structural basis for effector protein recognition by the Dot/Icm Type IVB coupling protein complex

Hyunmin Kim et al.

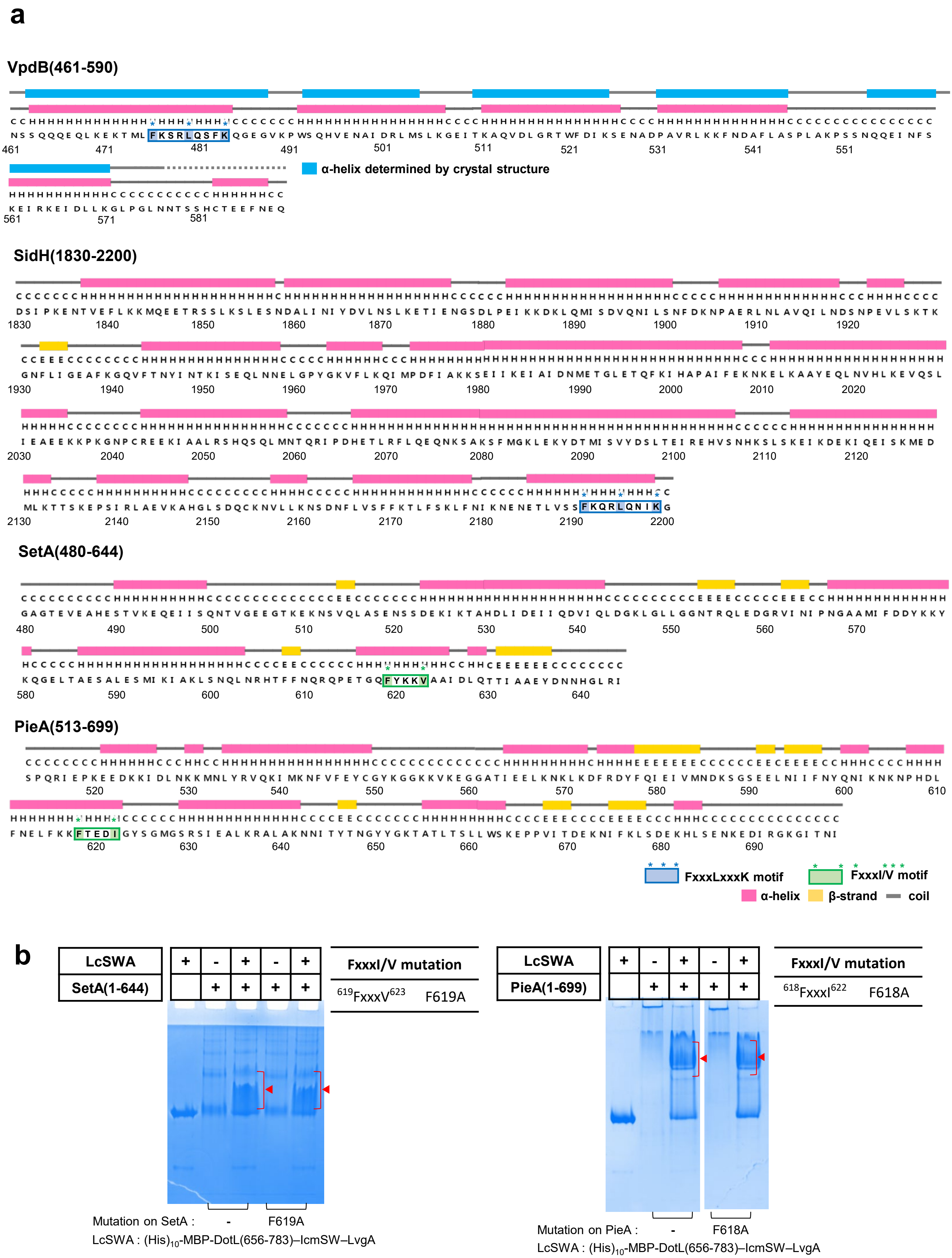
a
VpdB(461-590)
α-helix determined by crystal structure
SidH(1830-2200)
SetA(480-644)
PieA(513-699)
FxxxLxxxK motif
FxxxI/V motif
α-helix
β-strand
coil
b
LcSWA
SetA(1-644)
FxxxI/V mutation
^{619}FxxxV^{623} F619A
Mutation on SetA : - F619A
LcSWA : (His)$_{10}$-MBP-DotL(656-783)–IcmSW–LvgA
LcSWA
PieA(1-699)
FxxxI/V mutation
^{618}FxxxI^{622} F618A
Mutation on PieA : - F618A
LcSWA : (His)$_{10}$-MBP-DotL(656-783)–IcmSW–LvgA

Supplementary Figure 1. Secondary structure prediction, sequence motifs and protein-binding assay

(a) Secondary structure prediction and the positions of the sequence motifs.

Psi-PRED was used to predict the secondary structures of the four indicated protein constructs that bind to DotL(656-783)–IcmSW–LvgA. Experimentally determined secondary structures are shown for VpdB(461-590). The positions of the FxxxLxxxK and FxxxI/V sequence motifs are indicated by the blue or green boxes.

(b) Phe-to-Ala mutation in the FxxxI/V motif of SetA(480-644) and PieA(513-699).

The mutation points are indicated. The wild-type or mutant constructs were incubated with $(His)_{10}$-DotL(656-783)–IcmSW–LvgA complex (10 μM) at a 1:1 molar ratio, and subjected to native PAGE. No detectable change in the formation of the new protein band (red arrowheads) was observable for both of the protein constructs. The native PAGE analyses were repeated more than three times.

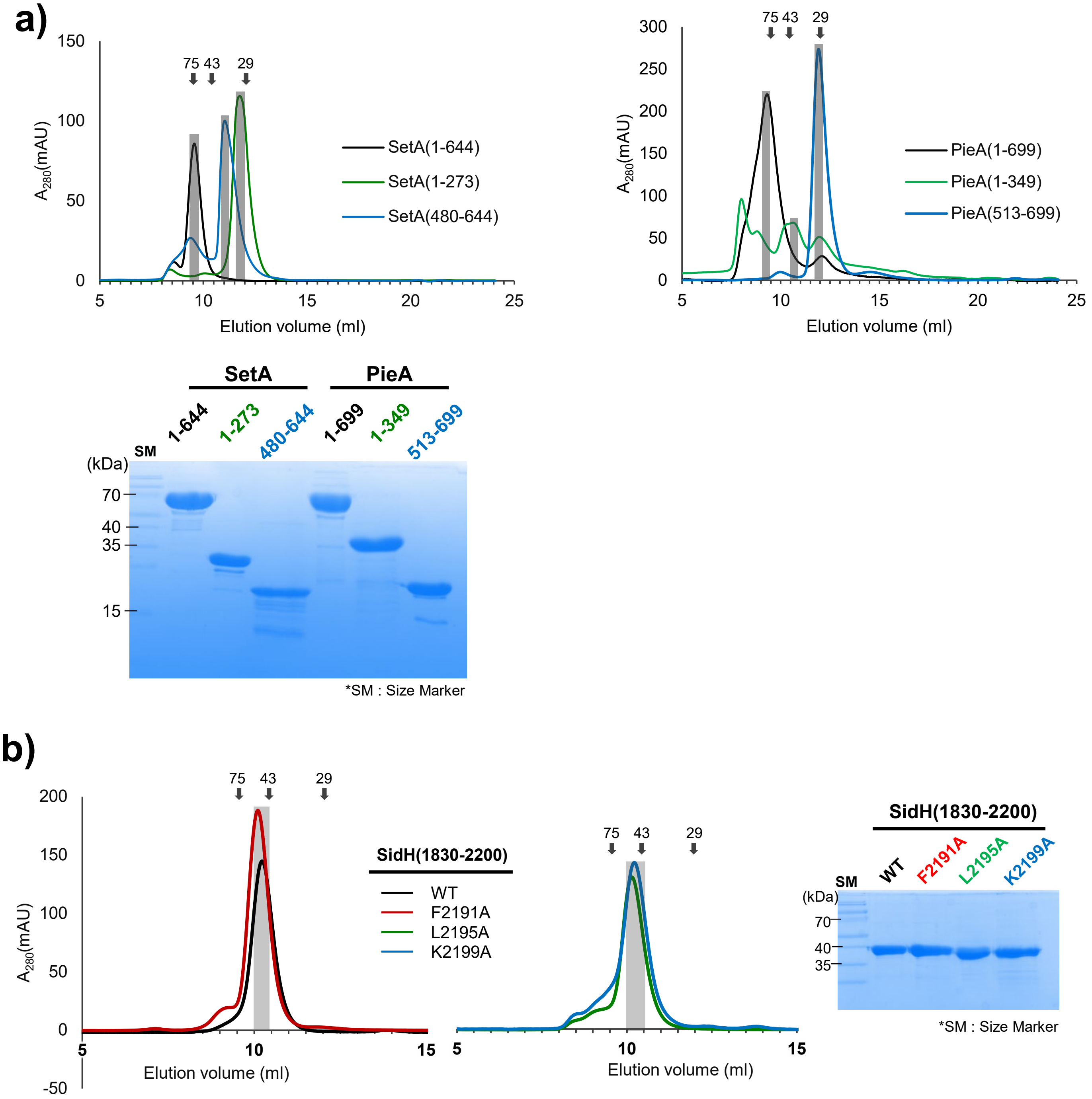

Supplementary Figure 2. Size-exclusion chromatography and SDS-PAGE of SetA, PieA and SidH constructs

The proteins were loaded onto a Superdex 75 Increase 10/300 GL column and eluted with a buffer solution containing 20 mM Tris-HCl (pH 7.5) plus 100 mM NaCl or 300 mM NaCl. Size marker positions are shown. The subfractions indicated by the grey bar were used for the SDS-PAGE runs and for all other biochemical analyses.

(a) Except for the PieA(1-349), the other constructs were eluted as a narrow major peak. SetA(480-644) was eluted as if it is a dimer, while all the others as a monomer.

(b) Wild-type SidH(1830-2200) and three other SidH mutants exhibited virtually the same elution peak, while minor higher-molecular weight species were observed with the mutants.

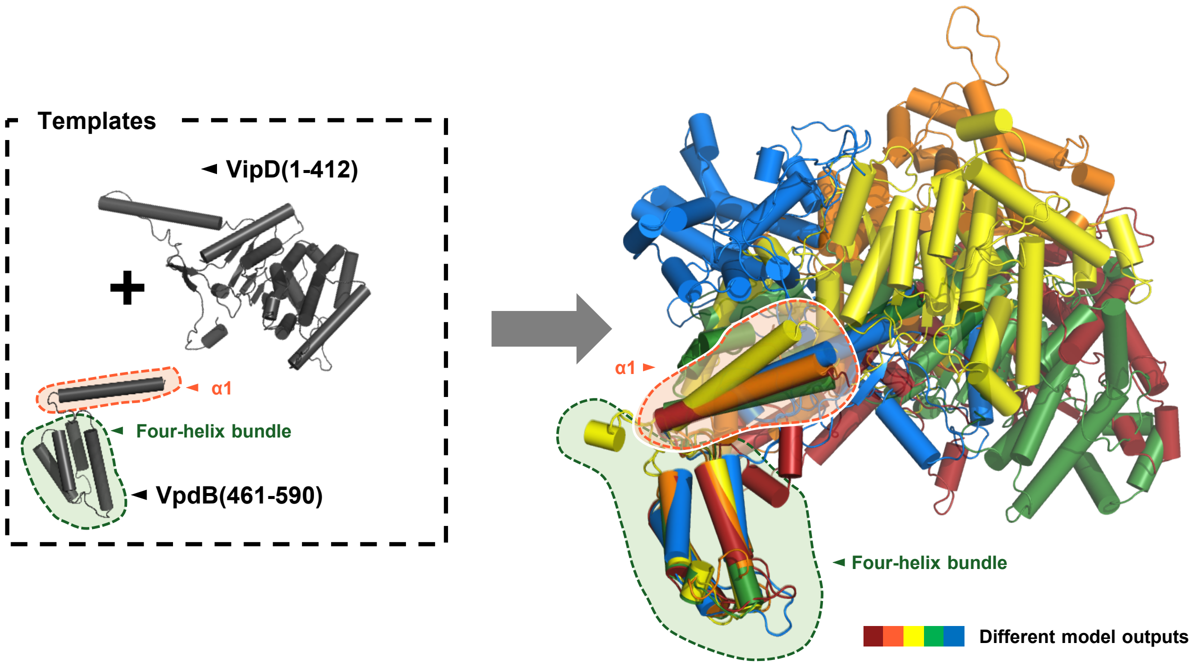

Supplementary Figure 3. Superposition of five homology models of full-length VpdB.

The two templates *(Left)* do not cover a middle segment of VpdB (residues 425-460), and consequently the homology models (*Right*) are heterogeneous in the relative orientations of the N- and C-terminal domains. The five output models are superposed with the C-terminal domains, which correspond to VpdB(461-590).

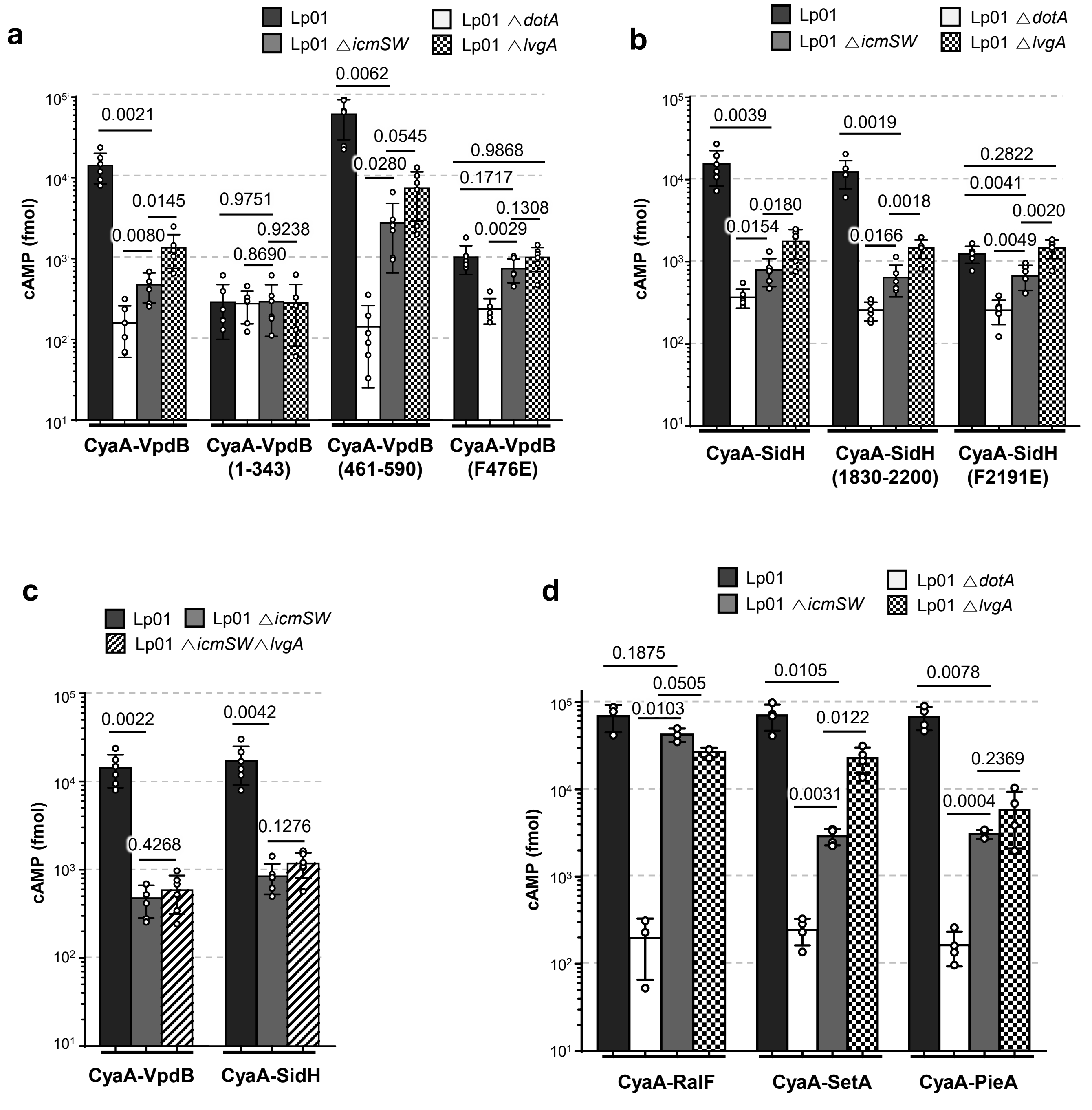

Supplementary Figure 4. Figure 4 with exact p-values marked on the graphs.

Supplementary Table 1. X-ray data collection and structure refinement statistics

**Data Collection**	**DotL(656-783)–IcmSW–LvgA–VpdB(461-590)**
Space group	P41212
Unit cell dimensions	
a, b, c (Å)	188.992, 188.992, 170.252
α, β, γ (°)	90, 90, 90
Wavelength (Å)	1.0000
Resolution (Å)	45.84-2.801 (2.901-2.801)[a]
R_{sym}	10.6(31.1)[a]
$I/\sigma(I)$	12.17(2.67)[a]
Completeness (%)	99.5(98.7)[a]
Redundancy	15.4(7.7)[a]
**Refinement**	
Resolution (Å)	47.25-2.801 (2.901-2.801)[a]
No. of reflections	75680
R_{work} / R_{free}	0.1990/0.2450
R.m.s deviations	
bond (Å) / angle (º)	0.009/1.014
Average B-values ($Å^2$)	36.65
Ramachandran plot (%)	
Favored / Additional allowed	90.7/9.0
Generously allowed	0.3

[a]The numbers in parentheses are the statistics from the highest resolution shell.

Supplementary Table 2. List of primers used in this study

Gene	Construct	F/R	Primer sequence	Vectors
**DotL**	(656-783)	F	ATACATATGGGTCAAAATGAACCCGAGCCT	pET 22b
		R	ATAGTCGACTGTTAATTCCTCTGCAGCCTTTTTATTGG	pET 22b
**IcmS**	(1-114)	F	ATACATATGGAGCGAGATATTAGCAAGTGTATGGC	pET 22b
		R	TATGGTACCCTAATCATACATTAACTCATCCAGGGGAGTATAA	pET 22b
**IcmW**	(1-151)	F	ATACATATGCCTGATTTAAGCCATGAAGCCT	pET 22b
		R	AATGTCGACTTATTCATCCCCTTCGAGTGCTCGTAAAAC	pET 22b
**LvgA**	(1-208)	F	AATCATATGGCAGACGGCGATATCGAAATC	pET 22b
		R	ATTAAGCTTTCATTTTCGTGCAGTAGTTCCAGAAGATACTGG	pET 22b
	*(I153E)	F	AGAAAGGCTTTTGAGTCGATTGAAAATTTACCTTATAACGTTGTTG TGA	pET 22b
		R	TCACAACAACGTTATAAGGTAAATTTTCAATCGACTCAAAAGCCTT TCT	pET 22b
**VpdB**	(11-590)	F	ATACATATGTCAGCAGAGCCATCTGTAAATCTAGGA	pET 22b
		R	ATAGTCGACCTGCTCATTAAACTCTTCTGTACAAT	pET 22b
	(11-488)	F	ATACATATGTCAGCAGAGCCATCTGTAAATCTAGGA	pET 22b
		R	ATAGTCGACTCATCCCTCTCCTTGTTTAAAACTTTGCAATCTTGATT TAAATA	pET 22b
	(11-343)	F	ATACATATGTCAGCAGAGCCATCTGTAAATCTAGGA	pET 22b
		R	ATAGTCGACTCAAGAAACCCCAATTGTAGTTACAACGCCTGGAGT ATA	pET 22b
	(425-590)	F	ATACATATGGGCGTGATCGAGGTAGGTAAAG	pET 22b
		R	ATAGTCGACCTGCTCATTAAACTCTTCTGTACAAT	pET 22b
	(461-590)	F	ATACATATGAACAGCAGTCAGCAACAAGAGCA	pET 22b
		R	ATAGTCGACCTGCTCATTAAACTCTTCTGTACAAT	pET 22b
	(461-488)	F	ATACATATGAACAGCAGTCAGCAACAAGAGCA	pET 22b
		R	ATAGTCGACTCCCTCTCCTTGTTTAAAACTTTGCAATCTTGATTTAA ATA	pET 22b
	(489-590)	F	ATACATATGGTCAAACCTTGGTCTCAGCATGTGGA	pET 22b
		R	ATAGTCGACCTGCTCATTAAACTCTTCTGTACAAT	pET 22b
	*(F476E)	F	AATTGAAAGAAAAGACCATGTTAGAAAAATCAAGATTGCAAAGTT	pET 22b
		R	AACTTTGCAATCTTGATTTTTCTAACATGGTCTTTTCTTTCAATT	pET 22b
	*(K470A)	F	AGAGCAATTGGCGGAAAAGACCAT	pET 22b
		R	ATGGTCTTTTCCGCCAATTGCTCT	pET 22b
	*(T473A)	F	TTGAAAGAAAAGGCCATGTTATTTA	pET 22b
		R	TAAATAACATGGCCTTTTCTTTCAA	pET 22b
	*(F476A)	F	AAGACCATGTTAGCGAAATCAAGATT	pET 22b
		R	AATCTTGATTTCGCTAACATGGTCTT	pET 22b

	*(L480A)	F	TTAAATCAAGAGCGCAAAGTTTTA	pET 22b
		R	TAAAACTTTGCGCTCTTGATTTAA	pET 22b
	*(F483A)	F	ATTGCAAAGTGCGAAACAAGGA	pET 22b
		R	TCCTTGTTTCGCACTTTGCAAT	pET 22b
	*(K484A)	F	TGCAAAGTTTTGCACAAGGAGA	pET 22b
		R	TCTCCTTGTGCAAAACTTTGCA	pET 22b
	(1-598)	F	GCGCAGTGGAACGCGGTACCATGAAAACGGTAAATAGTCAAAATGGTA	pMMB 207
		R	GCAGGTCGACTCTAGATTACGCCCTATATAGGGATGGTG	pMMB 207
	(1-343)	F	GCGCAGTGGAACGCGGTACCATGAAAACGGTAAATAGTCAAAATGGTA	pMMB 207
		R	GCAGGTCGACTCTAGAGCAAGAAACCCCAATTGTAGTTACAACG	pMMB 207
	(461-598)	F	GCGCAGTGGAACGCGGTACCAACAGCAGTCAGCAACAAGAGC	pMMB 207
		R	GCAGGTCGACTCTAGATTACGCCCTATATAGGGATGGTG	pMMB 207
**SetA**	(1-644)	F	ATACATATGATGTATAAAATATATTCATATCTAGGTTGGAGAATTGAT	pET 22b
		R	AGGATCCCGCGTCGACTATTCTTAAACCATGATTGTTATCG	pET 22b
	(1-273)	F	ATACATATGATGTATAAAATATATTCATATCTAGGTTGGAGAATTGAT	pET 22b
		R	ATAGTCGACTGTATTGTTCTGTGGTACATAAAAATAGT	pET 22b
	(480-644)	F	ATACATATGGGAGCGGGAACAGAAGTT	pET 22b
		R	AGGATCCCGCGTCGACTATTCTTAAACCATGATTGTTATCG	pET 22b
	*(F619A)	F	CAACCAGAAACCGGGCAAGCGTATAAAAAAGTGGCT	pET 22b
		R	AGCCACTTTTTTATACGCTTGCCCGGTTTCTGGTTG	pET 22b
	(1-644)	F	CGGTACCCGGGGATCCATGTATAAAATATATTCATATCTAG	pMMB 207
		R	CAAAACAGCCAAGCTTTTATATTCTTAAACCATGATTG	pMMB 207
**PieA**	(1-699)	F	AATCATATGCAAGAAAAAATTATCAACTTAGGGAAAGGGCT	pET 22b
		R	ATTTCTAGAGATATTCGTAATTCCTTTTCCGCGAATATCTTCC	pET 22b
	(1-349)	F	AATCATATGCAAGAAAAAATTATCAACTTAGGGAAAGGGCT	pET 22b
		R	ATAGTCGACCGACTTATCCAAACAGCGCTCAA	pET 22b
	(513-699)	F	AAGGAGATATACATATGTCACCACAGAGAATAGAACCAAAAG	pET 22b
		R	ATTTCTAGAGATATTCGTAATTCCTTTTCCGCGAATATCTTCC	pET 22b
	*(F618A)	F	TTAATGAGCTCTTTAAAAAAGCGACCGAAGACATAGGATATTCT	pET 22b
		R	AGAATATCCTATGTCTTCGGTCGCTTTTTTAAAGAGCTCATTAA	pET 22b
	(1-699)	F	GCGCAGTGGAACGCGGTATGCAAGAAAAAATTATCAACTTAG	pMMB 207
		R	GCAGGTCGACTCTAGACTAGATATTCGTAATTCCTTTTCCG	pMMB 207
**SidH**	(1830-2200)	F	ATAGTCGACGACAGTATTCCTAAAGAAAATACAGTGGAAT	pET 22b
		R	ATATCTAGAACCCTTGATATTTTGCAGCCTTT	pET 22b
	*(F2191A)	F	AATGAAACTTTGGTTTCATCAGCCAAACAAAGGCTGCAA	pET 22b

	R	TTGCAGCCTTTGTTTGGCTGATGAAACCAAAGTTTCATT	pET 22b
*(L2195A)	F	TCATCATTCAAACAAAGGGCCCAAAATATCAAGGGT	pET 22b
	R	ACCCTTGATATTTTGGGCCCTTTGTTTGAATGATGA	pET 22b
*(K2199A)	F	AACAAAGGCTGCAAAATATCGCCGGTTCTAGAGGAAAGCTTTAAT	pET 22b
	R	ATTAAAGCTTTCCTCTAGAACCGGCGATATTTTGCAGCCTTTGTT	pET 22b
(2183-2200)	F	CCATATGGGTGTCGACGAAAATGAAACTTTGGT	pET 22b
	R	ATATCTAGAACCCTTGATATTTTGCAGCCTTT	pET 22b
(1-2225)	F	CGGTACCCGGGGATCCATGAAAAGAACCATTGAAACCTACATCAT	pMMB 207
	R	CAAAACAGCCAAGCTTTTAGAATCTGGTAATATTGGCATTCACTAA AGGT	pMMB 207
(1830-2200)	F	CGGTACCCGGGGATCCGACAGTATTCCTAAAGAAAATACAGTGGA ATTCCTTA	pMMB 207
	R	CAAAACAGCCAAGCTTACCCTTGATATTTTGCAGCCTTTGT	pMMB 207

*Primers listed for constructs with point mutation are used for site-directed mutagenesis.
