# Supplementary material for: Structural basis for effector protein recognition by the Dot/Icm Type IVB coupling protein complex
Source: Nat Commun. 2020 May 26;11:2623. doi: 10.1038/s41467-020-16397-0 (PMC7251119; doi:10.1038/s41467-020-16397-0)
Supplement: Supplementary file 3 — Reporting Summary [file 41467_2020_16397_MOESM3_ESM.pdf]

nature research

Corresponding author(s): Byung-ha Oh

Last updated by author(s): Apr 17, 2020

Reporting Summary

Nature Research wishes to improve the reproducibility of the work that we publish. This form provides structure for consistency and transparency in reporting. For further information on Nature Research policies, see Authors & Referees and the Editorial Policy Checklist .

Statistics

For all statistical analyses, confirm that the following items are present in the figure legend, table legend, main text, or Methods section.

n/a	Confirmed	
x		The exact sample size (*n*) for each experimental group/condition, given as a discrete number and unit of measurement
x		A statement on whether measurements were taken from distinct samples or whether the same sample was measured repeatedly
	x	The statistical test(s) used AND whether they are one- or two-sided<br>*Only common tests should be described solely by name; describe more complex techniques in the Methods section.*
x		A description of all covariates tested
x		A description of any assumptions or corrections, such as tests of normality and adjustment for multiple comparisons
	x	A full description of the statistical parameters including central tendency (e.g. means) or other basic estimates (e.g. regression coefficient) AND variation (e.g. standard deviation) or associated estimates of uncertainty (e.g. confidence intervals)
	x	For null hypothesis testing, the test statistic (e.g. *F*, *t*, *r*) with confidence intervals, effect sizes, degrees of freedom and *P* value noted<br>*Give P values as exact values whenever suitable.*
x		For Bayesian analysis, information on the choice of priors and Markov chain Monte Carlo settings
x		For hierarchical and complex designs, identification of the appropriate level for tests and full reporting of outcomes
x		Estimates of effect sizes (e.g. Cohen's *d*, Pearson's *r*), indicating how they were calculated

Our web collection on statistics for biologists contains articles on many of the points above.

Software and code

Policy information about availability of computer code

Data collection	The X-ray diffraction data were collected at the beamline NE-3A at the Photon Factory, Japan.
Data analysis	X-ray diffraction data were processed with HKL2000 suite, and the structure was determined by using PHENIX(ver 1.10.1), COOT(ver 0.8.9.2) and CNS suites. Bio-layer interferometry data were analyzed by using Blitz Pro 1.1 software. CyaA translocation assay data were analyzed with Prism 7.0.

For manuscripts utilizing custom algorithms or software that are central to the research but not yet described in published literature, software must be made available to editors/reviewers. We strongly encourage code deposition in a community repository (e.g. GitHub). See the Nature Research guidelines for submitting code & software for further information.

Data

Policy information about availability of data

All manuscripts must include a data availability statement. This statement should provide the following information, where applicable:

- Accession codes, unique identifiers, or web links for publicly available datasets
- A list of figures that have associated raw data
- A description of any restrictions on data availability

The coordinates of the structure of DotL(656-783)–IcmSW–LvgA–VpdB(461-590) are deposited in the Protein Data Bank (PDB code: 7BWK). The PDB accession code for the coordinates used for phase determination is 5X90. N-terminal portion of the VipD structure (PDB code: 4AKF) was used for homology modeling.

Field-specific reporting

Please select the one below that is the best fit for your research. If you are not sure, read the appropriate sections before making your selection.

Life sciences study design

All studies must disclose on these points even when the disclosure is negative.

Sample size	The sample-size was determined regarding the same experiments previously reported in the field.
Data exclusions	No data was excluded.
Replication	All the presented experimental results were reliably reproduced, repeated more than at least two times independently.
Randomization	Random allocation was not relevant to the described experiments since no animal nor human studies are involved.
Blinding	Blinding group allocation was not relevant to the study since no animal nor human studies are involved.

Reporting for specific materials, systems and methods

We require information from authors about some types of materials, experimental systems and methods used in many studies. Here, indicate whether each material, system or method listed is relevant to your study. If you are not sure if a list item applies to your research, read the appropriate section before selecting a response.

Materials & experimental systems

n/a	Involved in the study	
☐	☒	Antibodies
☐	☒	Eukaryotic cell lines
☒	☐	Palaeontology
☒	☐	Animals and other organisms
☒	☐	Human research participants
☒	☐	Clinical data

Methods

n/a	Involved in the study	
☒	☐	ChIP-seq
☒	☐	Flow cytometry
☒	☐	MRI-based neuroimaging

Antibodies

Antibodies used	Anti-CyaA mouse monoclonal IgG (sc-13582, Santa Cruz Biotechnology, Inc.)<br>goat anti-mouse IgG (#31430, Invitrogen)<br>Rabbit anti-Legionella (raised by and gift from BioAcademia (Ibaragi, Osaka))
Validation	Anti-CyaA mouse monoclonal IgG (sc-13582) is raised against amino acids 1-400 of Bordetella pertussis CyaA, validated by WB analysis of authentic adenylate cyclase toxin and is applicable for WB. Goat anti-mouse IgG (#31430, Invitrogen) has species reactivity against mouse, probed with WB and is applicable for ELISA, IHC, IP, WB and ICC. Rabbit anti-Legionella antibody was validated by BioAcademia and is applicable for Legionella opsonization.

Eukaryotic cell lines

Policy information about cell lines

Cell line source(s)	CHO-FcR cell line was obtained from Craig Roy's Lab (Nagai et al. PNAS 2004, www.pnas.org/cgi/doi/10.1073/pnas.0406239101)
Authentication	The cell line was not authenticated.
Mycoplasma contamination	The cell line was not Mycoplasma tested.
Commonly misidentified lines<br>(See ICLAC register)	No commonly misidentified cell lines was used in this study.
